# 3′,6′-Bis(ethyl­amino)-2-[(2-hydroxy­ethyl)­amino]-2′,7′-dimethylspiro­[isoindoline-1,9′-xanthen]-3-one

**DOI:** 10.1107/S1600536808023611

**Published:** 2008-07-31

**Authors:** Mao-Zhong Tian, Xiao-Jun Peng

**Affiliations:** a School of Chemistry and Chemical Engineering, Shanxi Datong University, Datong 037009, People’s Republic of China; b State Key Laboratory of Fine Chemicals, Dalian University of Technology, 158 Zhongshan Rd., Dalian 116012, People’s Republic of China

## Abstract

In the title compound, C_28_H_32_N_4_O_3_, the dihedral angle between the planes of the xanthene ring system and the spiro­lactam ring is 85.99 (3)°. Mol­ecules are linked by inter­molecular O—H⋯O and N—H⋯O hydrogen-bonding inter­actions.

## Related literature

For the synthesis and related structures of rhodamine dyes, see: Ko *et al.* (2006 ▶); Wu *et al.* (2007 ▶); Zhang *et al.* (2008 ▶). For related literature on the photophysical properties and applications of rhodamine dyes, see: Lakowicz (2006 ▶).
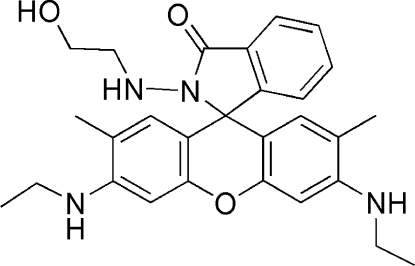


## Experimental

### 

#### Crystal data

C_28_H_32_N_4_O_3_
*M* *_r_* = 472.58
Triclinic, 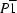

*a* = 9.3195 (18) Å
*b* = 9.4770 (16) Å
*c* = 15.384 (3) Å
α = 94.722 (18)°
β = 107.592 (13)°
γ = 98.924 (13)°
*V* = 1267.4 (4) Å^3^
*Z* = 2
Mo *K*α radiation
μ = 0.08 mm^−1^
*T* = 298 (2) K
0.30 × 0.20 × 0.15 mm

#### Data collection

Bruker APEXII CCD area-detector diffractometer
Absorption correction: none
8770 measured reflections
4305 independent reflections
3144 reflections with *I* > 2σ(*I*)
*R*_int_ = 0.024

#### Refinement

*R*[*F* ^2^ > 2σ(*F* ^2^)] = 0.045
*wR*(*F* ^2^) = 0.121
*S* = 1.06
4305 reflections
325 parameters
4 restraints
H atoms treated by a mixture of independent and constrained refinement
Δρ_max_ = 0.34 e Å^−3^
Δρ_min_ = −0.20 e Å^−3^

### 

Data collection: *APEX2* (Bruker 2005 ▶); cell refinement: *APEX2*; data reduction: *APEX2*; program(s) used to solve structure: *SHELXTL* (Sheldrick, 2008 ▶); program(s) used to refine structure: *SHELXTL*; molecular graphics: *SHELXTL*; software used to prepare material for publication: *SHELXTL*.

## Supplementary Material

Crystal structure: contains datablocks global, I. DOI: 10.1107/S1600536808023611/zl2122sup1.cif

Structure factors: contains datablocks I. DOI: 10.1107/S1600536808023611/zl2122Isup2.hkl

Additional supplementary materials:  crystallographic information; 3D view; checkCIF report

## Figures and Tables

**Table 1:** Hydrogen-bond geometry (Å, °)

*D*—H⋯*A*	*D*—H	H⋯*A*	*D*⋯*A*	*D*—H⋯*A*
N3—H3*A*⋯O3^i^	0.898 (16)	2.185 (18)	3.044 (2)	160 (2)
O3—H3*C*⋯O1^ii^	0.82	1.98	2.770 (2)	162

Symmetry codes: (i) 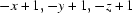; (ii) 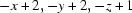.

**Fig. 1.**
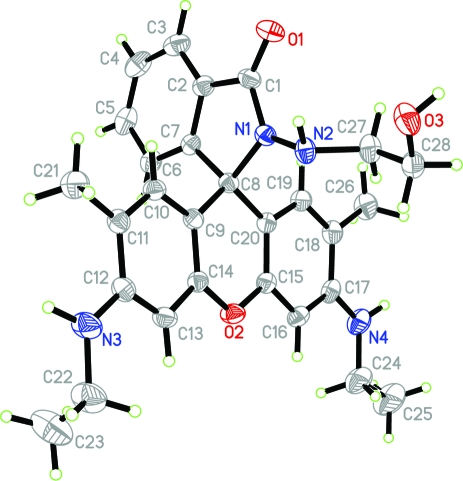
The structure of the title compound, showing the atom-numbering scheme and displacement ellipsoids at the 30% probability level. H atoms are represented as small spheres of arbitrary radius.

**Fig. 2.**
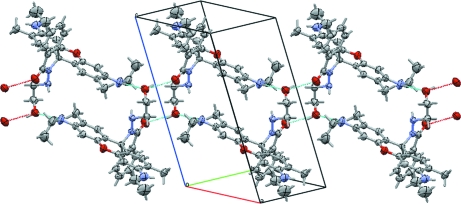
The molecular packing of the title compound showing the double stranded hydrogen bond connected chains. Dashed lines indicate hydrogen bonds.

## supplementary crystallographic information

### Comment

Among many fluorescent compounds, rhodamine dyes are known to have excellent photophysical properties, (Lakowicz, 2006) and they are one of the most widely used fluorophores for labeling and sensing biomolecules (Ko *et al.*, 2006; Wu *et al.*, 2007). There are a few single-crystal reports about rhodamine derivatives bearing a lactam moiety (Wu *et al.*, 2007; Zhang *et al.*, 2008). Detailed information on their molecular and crystal structures is necessary to understand their photophysical and photochemical properties.

In agreement with other reported models, (Wu *et al.*, 2007) the main skeleton of the title molecule is formed by the xanthene ring and the spirolactam-ring. As shown in Figure 1, the atoms of the xanthene ring and spirolactam-rings are both nearly planar and are almost perpendicular to each other. R.m.s. deviations from planarity are 0.028 (1) Å for the xanthene ring and 0.033 (0) Å for the spirolactam-ring, respectively. The dihedral angle between the planes of the xanthene ring and the spirolactam ring is 85.99 (3)°.

Analysis of the crystal packing of the title molecule (Figure 2), shows that the molecules of the title compound are connected *via* intermolecular N3—H3A···O3 and O3—H3C···O1 hydrogen bonds (Table 1). The oxygen atom on the spirolactam-ring acts as acceptor for an O—H···O hydrogen bond from a neighboring molecule. The oxygen atom of the hydroxyl group in turn acts as acceptor for a N—H···O hydrogen bond from again another molecule, thus forming a chain with two consecutive hydrogen bonds of the type N—H···O—H···O═C. Via these hydrogen bonds molecules are connected into double stranded chains as shown in Figure 2.

### Experimental

Sodium borohydride (15.2 mg, 0.4 mmol) was slowly added to a solution of 3',6'-bis(ethylamino)-2',7'-dimethyl-2-(2-oxoethylideneamino)spiro [isoindoline-1,9'-xanthen]-3-one (132 mg, 0.3 mmol) in ethanol (20 ml). The reaction mixture was stirred for 2 h at room temperature and the solvent was totally removed under reduced pressure. The crude product was dissolved in CH_2_Cl_2_ (20 ml) and 3 ml of an aqueous solution of K_2_CO_3_ was added. The organic layer was separated and dried over MgSO_4_. After filtration, the solvent was removed under reduced pressure. The residue was placed on a silica gel column (200–300 mesh). The column was eluted with a mixture (2:1, *v/v*) of petroleum ether /ethyl acetate, to give 131.5 mg of the title compound (93%). Crystals were grown by dissolving the compound in CH_2_Cl_2_ and slowly diffusing n-hexane into the solution.

### Refinement

Geometrically constrained hydrogen atoms were placed in calculated positions and refined using the riding model (C—H = 0.93-0.96 Å, and O—H = 0.82 Å), with *U*_iso_(H) = 1.2*U*_eq_(C) or 1.5*U*_eq_(methyl C, O). All amine hydrogen atoms were located in difference density Fourier maps, were introduced with a distance restraint (N—H = 0.89 (2) Å) and refined freely. The isotropic displacement parameter was set to *U*_iso_(H) = 1.2*U*_eq_(N).

### Crystal data

**Table:**

C_28_H_32_N_4_O_3_	*Z* = 2
*M**_r_* = 472.58	*F*_000_ = 504
Triclinic, *P*1	*D*_x_ = 1.238 Mg m^−^^3^
Hall symbol: -P 1	Mo *K*α radiation λ = 0.71073 Å
*a* = 9.3195 (18) Å	Cell parameters from 2583 reflections
*b* = 9.4770 (16) Å	θ = 2.3–26.8º
*c* = 15.384 (3) Å	µ = 0.08 mm^−^^1^
α = 94.722 (18)º	*T* = 298 (2) K
β = 107.592 (13)º	Block, colourless
γ = 98.924 (13)º	0.30 × 0.20 × 0.15 mm
*V* = 1267.4 (4) Å^3^	

### Data collection

**Table:**

Bruker APEXII CCD area-detector diffractometer	3144 reflections with *I* > 2σ(*I*)
Radiation source: fine-focus sealed tube	*R*_int_ = 0.024
Monochromator: graphite	θ_max_ = 25.0º
*T* = 298(2) K	θ_min_ = 2.2º
φ and ω scans	*h* = −11→11
Absorption correction: none	*k* = −11→11
8770 measured reflections	*l* = −18→18
4305 independent reflections	

### Refinement

**Table:**

Refinement on *F*^2^	Secondary atom site location: difference Fourier map
Least-squares matrix: full	Hydrogen site location: inferred from neighbouring sites
*R*[*F*^2^ > 2σ(*F*^2^)] = 0.045	H atoms treated by a mixture of independent and constrained refinement
*wR*(*F*^2^) = 0.121	*w* = 1/[σ^2^(*F*_o_^2^) + (0.068*P*)^2^ + 0.1313*P*] where *P* = (*F*_o_^2^ + 2*F*_c_^2^)/3
*S* = 1.07	(Δ/σ)_max_ < 0.001
4305 reflections	Δρ_max_ = 0.34 e Å^−^^3^
325 parameters	Δρ_min_ = −0.19 e Å^−^^3^
4 restraints	Extinction correction: SHELXL, Fc^*^=kFc[1+0.001xFc^2^λ^3^/sin(2θ)]^-1/4^
Primary atom site location: structure-invariant direct methods	Extinction coefficient: 0.028 (8)

### Special details

**Table:**

Geometry. All esds (except the esd in the dihedral angle between two l.s. planes) are estimated using the full covariance matrix. The cell esds are taken into account individually in the estimation of esds in distances, angles and torsion angles; correlations between esds in cell parameters are only used when they are defined by crystal symmetry. An approximate (isotropic) treatment of cell esds is used for estimating esds involving l.s. planes.
Refinement. Refinement of F^2^ against ALL reflections. The weighted R-factor wR and goodness of fit S are based on F^2^, conventional R-factors R are based on F, with F set to zero for negative F^2^. The threshold expression of F^2^ > 2σ(F^2^) is used only for calculating R-factors(gt) etc. and is not relevant to the choice of reflections for refinement. R-factors based on F^2^ are statistically about twice as large as those based on F, and R- factors based on ALL data will be even larger.

### Fractional atomic coordinates and isotropic or equivalent isotropic displacement parameters (Å^2^)

**Table:**

	*x*	*y*	*z*	*U*_iso_*/*U*_eq_	
O2	0.69421 (15)	0.29618 (12)	0.25724 (9)	0.0457 (3)	
C16	0.8939 (2)	0.3280 (2)	0.19461 (12)	0.0433 (5)	
H16A	0.8966	0.2307	0.1971	0.052*	
C14	0.5894 (2)	0.35022 (18)	0.29116 (11)	0.0364 (4)	
C10	0.4573 (2)	0.52933 (18)	0.32506 (12)	0.0393 (4)	
H10A	0.4422	0.6240	0.3249	0.047*	
C9	0.5676 (2)	0.49027 (17)	0.28839 (11)	0.0346 (4)	
C20	0.7802 (2)	0.53260 (18)	0.22210 (11)	0.0353 (4)	
N1	0.73649 (16)	0.72826 (14)	0.32209 (9)	0.0371 (4)	
C8	0.66223 (19)	0.59937 (17)	0.25166 (11)	0.0342 (4)	
C15	0.7889 (2)	0.38924 (18)	0.22530 (11)	0.0374 (4)	
C13	0.5043 (2)	0.25175 (19)	0.32712 (13)	0.0441 (5)	
H13A	0.5219	0.1578	0.3280	0.053*	
C17	0.9953 (2)	0.4115 (2)	0.16000 (12)	0.0443 (5)	
C19	0.8840 (2)	0.6140 (2)	0.18778 (12)	0.0425 (4)	
H19A	0.8801	0.7111	0.1853	0.051*	
C11	0.3697 (2)	0.43659 (19)	0.36141 (12)	0.0410 (4)	
O1	0.72540 (17)	0.96667 (13)	0.35009 (10)	0.0621 (4)	
N4	1.0976 (2)	0.3528 (2)	0.12588 (12)	0.0589 (5)	
H4A	1.175 (2)	0.413 (2)	0.1209 (16)	0.071*	
C7	0.56703 (19)	0.67124 (18)	0.17571 (11)	0.0354 (4)	
C12	0.3930 (2)	0.29227 (19)	0.36189 (12)	0.0431 (5)	
C18	0.9910 (2)	0.5596 (2)	0.15757 (12)	0.0449 (5)	
N2	0.82046 (17)	0.70879 (16)	0.41102 (10)	0.0422 (4)	
H2A	0.787 (2)	0.7625 (19)	0.4486 (12)	0.051*	
C2	0.5870 (2)	0.81693 (19)	0.20269 (12)	0.0414 (4)	
N3	0.3075 (2)	0.19768 (18)	0.39963 (14)	0.0619 (5)	
H3A	0.216 (2)	0.216 (2)	0.3996 (16)	0.074*	
C21	0.2564 (3)	0.4877 (2)	0.40272 (15)	0.0576 (5)	
H21A	0.2557	0.5875	0.3962	0.086*	
H21B	0.2854	0.4766	0.4668	0.086*	
H21C	0.1559	0.4317	0.3714	0.086*	
C1	0.6898 (2)	0.85141 (18)	0.29889 (13)	0.0422 (4)	
C6	0.4744 (2)	0.6098 (2)	0.08895 (12)	0.0443 (5)	
H6A	0.4597	0.5111	0.0707	0.053*	
C26	1.1000 (3)	0.6534 (2)	0.12199 (15)	0.0609 (6)	
H26A	1.0818	0.7503	0.1259	0.091*	
H26B	1.0843	0.6173	0.0590	0.091*	
H26C	1.2035	0.6524	0.1584	0.091*	
C5	0.4036 (2)	0.6986 (3)	0.02931 (13)	0.0561 (6)	
H5A	0.3403	0.6588	−0.0297	0.067*	
C4	0.4252 (3)	0.8445 (3)	0.05583 (15)	0.0609 (6)	
H4B	0.3777	0.9021	0.0142	0.073*	
C3	0.5162 (3)	0.9066 (2)	0.14321 (15)	0.0565 (6)	
H3B	0.5299	1.0050	0.1617	0.068*	
C22	0.3242 (3)	0.0484 (2)	0.40404 (18)	0.0770 (7)	
H22A	0.2842	0.0130	0.4511	0.092*	
H22B	0.4325	0.0445	0.4227	0.092*	
C25	1.2242 (4)	0.1710 (4)	0.0779 (2)	0.1053 (11)	
H25A	1.2292	0.0705	0.0766	0.158*	
H25B	1.3230	0.2277	0.1123	0.158*	
H25C	1.1947	0.1941	0.0161	0.158*	
C24	1.1072 (3)	0.2038 (3)	0.12294 (18)	0.0750 (7)	
H24A	1.0077	0.1456	0.0886	0.090*	
H24B	1.1361	0.1791	0.1850	0.090*	
C23	0.2464 (4)	−0.0473 (3)	0.3172 (2)	0.1070 (11)	
H23A	0.2638	−0.1433	0.3251	0.160*	
H23B	0.2859	−0.0138	0.2703	0.160*	
H23C	0.1383	−0.0475	0.2995	0.160*	
C28	1.0649 (2)	0.7337 (2)	0.52897 (13)	0.0526 (5)	
H28A	1.0422	0.6325	0.5356	0.063*	
H28B	1.1749	0.7618	0.5430	0.063*	
C27	0.9862 (2)	0.7548 (2)	0.43216 (13)	0.0529 (5)	
H27A	1.0096	0.8557	0.4249	0.063*	
H27B	1.0219	0.6986	0.3901	0.063*	
O3	1.01619 (16)	0.81677 (16)	0.59124 (9)	0.0627 (4)	
H3C	1.0814	0.8896	0.6149	0.094*	

### Atomic displacement parameters (Å^2^)

**Table:**

	*U*^11^	*U*^22^	*U*^33^	*U*^12^	*U*^13^	*U*^23^
O2	0.0503 (8)	0.0360 (7)	0.0620 (8)	0.0138 (6)	0.0308 (7)	0.0100 (6)
C16	0.0461 (11)	0.0459 (10)	0.0416 (10)	0.0196 (9)	0.0147 (9)	0.0048 (8)
C14	0.0355 (10)	0.0375 (9)	0.0364 (9)	0.0076 (8)	0.0123 (8)	0.0030 (7)
C10	0.0409 (11)	0.0355 (9)	0.0440 (10)	0.0087 (8)	0.0167 (9)	0.0051 (8)
C9	0.0357 (10)	0.0342 (9)	0.0333 (9)	0.0050 (7)	0.0111 (8)	0.0036 (7)
C20	0.0347 (10)	0.0390 (9)	0.0325 (9)	0.0078 (8)	0.0112 (8)	0.0025 (7)
N1	0.0386 (9)	0.0354 (8)	0.0364 (8)	0.0039 (6)	0.0131 (7)	0.0015 (6)
C8	0.0358 (10)	0.0333 (9)	0.0336 (9)	0.0051 (7)	0.0130 (8)	0.0004 (7)
C15	0.0368 (10)	0.0412 (10)	0.0336 (9)	0.0080 (8)	0.0103 (8)	0.0037 (7)
C13	0.0489 (12)	0.0335 (9)	0.0525 (11)	0.0073 (8)	0.0196 (9)	0.0089 (8)
C17	0.0381 (11)	0.0623 (12)	0.0342 (10)	0.0176 (9)	0.0109 (8)	0.0021 (8)
C19	0.0421 (11)	0.0439 (10)	0.0447 (10)	0.0081 (8)	0.0184 (9)	0.0069 (8)
C11	0.0392 (11)	0.0430 (10)	0.0431 (10)	0.0052 (8)	0.0183 (9)	0.0043 (8)
O1	0.0694 (10)	0.0368 (7)	0.0717 (10)	0.0053 (7)	0.0168 (8)	−0.0085 (7)
N4	0.0516 (12)	0.0751 (13)	0.0613 (11)	0.0260 (10)	0.0282 (9)	0.0067 (9)
C7	0.0344 (10)	0.0409 (9)	0.0361 (9)	0.0091 (7)	0.0170 (8)	0.0082 (7)
C12	0.0442 (11)	0.0432 (10)	0.0431 (10)	0.0024 (8)	0.0181 (9)	0.0078 (8)
C18	0.0409 (11)	0.0559 (11)	0.0405 (10)	0.0086 (9)	0.0177 (9)	0.0042 (8)
N2	0.0365 (9)	0.0524 (9)	0.0341 (8)	0.0025 (7)	0.0100 (7)	0.0016 (7)
C2	0.0420 (11)	0.0412 (10)	0.0465 (11)	0.0110 (8)	0.0193 (9)	0.0103 (8)
N3	0.0665 (13)	0.0475 (10)	0.0872 (13)	0.0061 (9)	0.0472 (11)	0.0188 (9)
C21	0.0587 (14)	0.0569 (12)	0.0715 (14)	0.0131 (10)	0.0391 (12)	0.0143 (10)
C1	0.0452 (11)	0.0318 (9)	0.0529 (11)	0.0036 (8)	0.0229 (9)	0.0030 (8)
C6	0.0424 (11)	0.0547 (11)	0.0391 (10)	0.0133 (9)	0.0165 (9)	0.0034 (8)
C26	0.0521 (13)	0.0742 (14)	0.0654 (14)	0.0082 (11)	0.0337 (11)	0.0098 (11)
C5	0.0484 (13)	0.0871 (16)	0.0380 (11)	0.0237 (11)	0.0157 (9)	0.0102 (10)
C4	0.0665 (15)	0.0788 (16)	0.0540 (13)	0.0355 (12)	0.0269 (12)	0.0315 (11)
C3	0.0665 (14)	0.0491 (11)	0.0667 (14)	0.0234 (10)	0.0304 (12)	0.0209 (10)
C22	0.092 (2)	0.0648 (15)	0.0916 (19)	0.0074 (13)	0.0534 (16)	0.0264 (13)
C25	0.099 (2)	0.125 (2)	0.117 (2)	0.064 (2)	0.0545 (19)	−0.0001 (19)
C24	0.0710 (17)	0.0829 (17)	0.0834 (17)	0.0393 (14)	0.0322 (14)	0.0044 (13)
C23	0.139 (3)	0.0716 (18)	0.117 (3)	−0.0130 (18)	0.070 (2)	0.0006 (17)
C28	0.0421 (12)	0.0554 (12)	0.0539 (12)	0.0079 (9)	0.0088 (10)	−0.0005 (9)
C27	0.0392 (12)	0.0676 (13)	0.0495 (12)	0.0039 (10)	0.0148 (9)	0.0041 (9)
O3	0.0519 (9)	0.0710 (9)	0.0563 (9)	−0.0028 (7)	0.0162 (7)	−0.0104 (7)

### Geometric parameters (Å, °)

**Table:**

O2—C15	1.375 (2)	C2—C1	1.477 (3)
O2—C14	1.381 (2)	N3—C22	1.452 (3)
C16—C15	1.388 (2)	N3—H3A	0.898 (16)
C16—C17	1.394 (3)	C21—H21A	0.9600
C16—H16A	0.9300	C21—H21B	0.9600
C14—C9	1.376 (2)	C21—H21C	0.9600
C14—C13	1.388 (3)	C6—C5	1.385 (3)
C10—C11	1.373 (3)	C6—H6A	0.9300
C10—C9	1.395 (2)	C26—H26A	0.9600
C10—H10A	0.9300	C26—H26B	0.9600
C9—C8	1.510 (2)	C26—H26C	0.9600
C20—C15	1.378 (2)	C5—C4	1.375 (3)
C20—C19	1.397 (2)	C5—H5A	0.9300
C20—C8	1.512 (2)	C4—C3	1.378 (3)
N1—C1	1.346 (2)	C4—H4B	0.9300
N1—N2	1.399 (2)	C3—H3B	0.9300
N1—C8	1.487 (2)	C22—C23	1.464 (4)
C8—C7	1.519 (2)	C22—H22A	0.9700
C13—C12	1.391 (3)	C22—H22B	0.9700
C13—H13A	0.9300	C25—C24	1.514 (3)
C17—N4	1.383 (2)	C25—H25A	0.9600
C17—C18	1.413 (3)	C25—H25B	0.9600
C19—C18	1.370 (2)	C25—H25C	0.9600
C19—H19A	0.9300	C24—H24A	0.9700
C11—C12	1.418 (2)	C24—H24B	0.9700
C11—C21	1.506 (2)	C23—H23A	0.9600
O1—C1	1.229 (2)	C23—H23B	0.9600
N4—C24	1.426 (3)	C23—H23C	0.9600
N4—H4A	0.872 (16)	C28—O3	1.412 (2)
C7—C6	1.373 (2)	C28—C27	1.493 (3)
C7—C2	1.376 (2)	C28—H28A	0.9700
C12—N3	1.386 (2)	C28—H28B	0.9700
C18—C26	1.502 (3)	C27—H27A	0.9700
N2—C27	1.465 (2)	C27—H27B	0.9700
N2—H2A	0.892 (14)	O3—H3C	0.8200
C2—C3	1.390 (3)		
			
C15—O2—C14	118.45 (13)	H21A—C21—H21B	109.5
C15—C16—C17	120.39 (17)	C11—C21—H21C	109.5
C15—C16—H16A	119.8	H21A—C21—H21C	109.5
C17—C16—H16A	119.8	H21B—C21—H21C	109.5
C9—C14—O2	123.10 (16)	O1—C1—N1	125.40 (18)
C9—C14—C13	121.84 (16)	O1—C1—C2	128.51 (17)
O2—C14—C13	115.05 (15)	N1—C1—C2	106.06 (14)
C11—C10—C9	124.05 (16)	C7—C6—C5	118.23 (18)
C11—C10—H10A	118.0	C7—C6—H6A	120.9
C9—C10—H10A	118.0	C5—C6—H6A	120.9
C14—C9—C10	116.65 (16)	C18—C26—H26A	109.5
C14—C9—C8	122.29 (15)	C18—C26—H26B	109.5
C10—C9—C8	121.01 (14)	H26A—C26—H26B	109.5
C15—C20—C19	116.72 (16)	C18—C26—H26C	109.5
C15—C20—C8	122.27 (16)	H26A—C26—H26C	109.5
C19—C20—C8	120.99 (15)	H26B—C26—H26C	109.5
C1—N1—N2	124.66 (14)	C4—C5—C6	121.15 (19)
C1—N1—C8	114.93 (14)	C4—C5—H5A	119.4
N2—N1—C8	118.93 (13)	C6—C5—H5A	119.4
N1—C8—C9	110.24 (13)	C5—C4—C3	120.93 (19)
N1—C8—C20	111.05 (13)	C5—C4—H4B	119.5
C9—C8—C20	110.54 (13)	C3—C4—H4B	119.5
N1—C8—C7	99.20 (12)	C4—C3—C2	117.61 (19)
C9—C8—C7	113.69 (14)	C4—C3—H3B	121.2
C20—C8—C7	111.64 (13)	C2—C3—H3B	121.2
O2—C15—C20	123.17 (15)	N3—C22—C23	114.1 (2)
O2—C15—C16	115.15 (15)	N3—C22—H22A	108.7
C20—C15—C16	121.68 (17)	C23—C22—H22A	108.7
C14—C13—C12	120.58 (16)	N3—C22—H22B	108.7
C14—C13—H13A	119.7	C23—C22—H22B	108.7
C12—C13—H13A	119.7	H22A—C22—H22B	107.6
N4—C17—C16	121.86 (18)	C24—C25—H25A	109.5
N4—C17—C18	118.95 (18)	C24—C25—H25B	109.5
C16—C17—C18	119.18 (16)	H25A—C25—H25B	109.5
C18—C19—C20	124.05 (17)	C24—C25—H25C	109.5
C18—C19—H19A	118.0	H25A—C25—H25C	109.5
C20—C19—H19A	118.0	H25B—C25—H25C	109.5
C10—C11—C12	117.97 (16)	N4—C24—C25	110.7 (2)
C10—C11—C21	121.09 (16)	N4—C24—H24A	109.5
C12—C11—C21	120.90 (16)	C25—C24—H24A	109.5
C17—N4—C24	123.02 (19)	N4—C24—H24B	109.5
C17—N4—H4A	116.6 (15)	C25—C24—H24B	109.5
C24—N4—H4A	118.1 (15)	H24A—C24—H24B	108.1
C6—C7—C2	120.62 (16)	C22—C23—H23A	109.5
C6—C7—C8	128.39 (15)	C22—C23—H23B	109.5
C2—C7—C8	110.96 (15)	H23A—C23—H23B	109.5
N3—C12—C13	122.25 (17)	C22—C23—H23C	109.5
N3—C12—C11	118.83 (17)	H23A—C23—H23C	109.5
C13—C12—C11	118.89 (16)	H23B—C23—H23C	109.5
C19—C18—C17	117.97 (17)	O3—C28—C27	110.65 (16)
C19—C18—C26	121.36 (18)	O3—C28—H28A	109.5
C17—C18—C26	120.67 (17)	C27—C28—H28A	109.5
N1—N2—C27	113.12 (14)	O3—C28—H28B	109.5
N1—N2—H2A	105.2 (12)	C27—C28—H28B	109.5
C27—N2—H2A	109.7 (13)	H28A—C28—H28B	108.1
C7—C2—C3	121.43 (18)	N2—C27—C28	109.01 (16)
C7—C2—C1	108.54 (15)	N2—C27—H27A	109.9
C3—C2—C1	130.03 (17)	C28—C27—H27A	109.9
C12—N3—C22	122.85 (18)	N2—C27—H27B	109.9
C12—N3—H3A	117.2 (16)	C28—C27—H27B	109.9
C22—N3—H3A	115.2 (16)	H27A—C27—H27B	108.3
C11—C21—H21A	109.5	C28—O3—H3C	109.5
C11—C21—H21B	109.5		
			
C15—O2—C14—C9	4.9 (2)	C9—C8—C7—C6	−65.7 (2)
C15—O2—C14—C13	−176.33 (15)	C20—C8—C7—C6	60.2 (2)
O2—C14—C9—C10	179.82 (15)	N1—C8—C7—C2	−0.61 (17)
C13—C14—C9—C10	1.1 (3)	C9—C8—C7—C2	116.38 (16)
O2—C14—C9—C8	−2.9 (3)	C20—C8—C7—C2	−117.72 (16)
C13—C14—C9—C8	178.39 (15)	C14—C13—C12—N3	−178.99 (18)
C11—C10—C9—C14	−1.2 (3)	C14—C13—C12—C11	−1.1 (3)
C11—C10—C9—C8	−178.52 (16)	C10—C11—C12—N3	178.97 (18)
C1—N1—C8—C9	−115.45 (16)	C21—C11—C12—N3	1.2 (3)
N2—N1—C8—C9	51.36 (19)	C10—C11—C12—C13	1.0 (3)
C1—N1—C8—C20	121.68 (16)	C21—C11—C12—C13	−176.80 (17)
N2—N1—C8—C20	−71.50 (18)	C20—C19—C18—C17	1.0 (3)
C1—N1—C8—C7	4.12 (17)	C20—C19—C18—C26	−179.60 (17)
N2—N1—C8—C7	170.93 (14)	N4—C17—C18—C19	177.03 (17)
C14—C9—C8—N1	−124.27 (17)	C16—C17—C18—C19	−1.4 (3)
C10—C9—C8—N1	52.9 (2)	N4—C17—C18—C26	−2.4 (3)
C14—C9—C8—C20	−1.1 (2)	C16—C17—C18—C26	179.15 (17)
C10—C9—C8—C20	176.09 (14)	C1—N1—N2—C27	−86.1 (2)
C14—C9—C8—C7	125.37 (17)	C8—N1—N2—C27	108.48 (17)
C10—C9—C8—C7	−57.4 (2)	C6—C7—C2—C3	−0.8 (3)
C15—C20—C8—N1	125.88 (17)	C8—C7—C2—C3	177.32 (17)
C19—C20—C8—N1	−55.7 (2)	C6—C7—C2—C1	179.23 (16)
C15—C20—C8—C9	3.2 (2)	C8—C7—C2—C1	−2.6 (2)
C19—C20—C8—C9	−178.44 (15)	C13—C12—N3—C22	−1.5 (3)
C15—C20—C8—C7	−124.42 (17)	C11—C12—N3—C22	−179.4 (2)
C19—C20—C8—C7	54.0 (2)	N2—N1—C1—O1	6.8 (3)
C14—O2—C15—C20	−2.7 (2)	C8—N1—C1—O1	172.78 (17)
C14—O2—C15—C16	178.35 (14)	N2—N1—C1—C2	−171.77 (15)
C19—C20—C15—O2	−179.92 (15)	C8—N1—C1—C2	−5.82 (19)
C8—C20—C15—O2	−1.5 (3)	C7—C2—C1—O1	−173.45 (19)
C19—C20—C15—C16	−1.0 (2)	C3—C2—C1—O1	6.6 (3)
C8—C20—C15—C16	177.45 (15)	C7—C2—C1—N1	5.1 (2)
C17—C16—C15—O2	179.53 (15)	C3—C2—C1—N1	−174.85 (19)
C17—C16—C15—C20	0.5 (3)	C2—C7—C6—C5	0.8 (3)
C9—C14—C13—C12	0.0 (3)	C8—C7—C6—C5	−177.00 (17)
O2—C14—C13—C12	−178.83 (15)	C7—C6—C5—C4	0.1 (3)
C15—C16—C17—N4	−177.69 (16)	C6—C5—C4—C3	−1.0 (3)
C15—C16—C17—C18	0.7 (3)	C5—C4—C3—C2	1.0 (3)
C15—C20—C19—C18	0.2 (3)	C7—C2—C3—C4	−0.1 (3)
C8—C20—C19—C18	−178.24 (16)	C1—C2—C3—C4	179.86 (19)
C9—C10—C11—C12	0.2 (3)	C12—N3—C22—C23	−79.0 (3)
C9—C10—C11—C21	177.93 (17)	C17—N4—C24—C25	177.3 (2)
C16—C17—N4—C24	0.3 (3)	N1—N2—C27—C28	178.42 (14)
C18—C17—N4—C24	−178.14 (19)	O3—C28—C27—N2	−60.6 (2)
N1—C8—C7—C6	177.36 (17)		

### Hydrogen-bond geometry (Å, °)

**Table:**

*D*—H···*A*	*D*—H	H···*A*	*D*···*A*	*D*—H···*A*
N3—H3A···O3^i^	0.898 (16)	2.185 (18)	3.044 (2)	160 (2)
O3—H3C···O1^ii^	0.82	1.98	2.770 (2)	162

Symmetry codes: (i) −*x*+1, −*y*+1, −*z*+1; (ii) −*x*+2, −*y*+2, −*z*+1.

## References

[bb1] Bruker (2005). *APEX2* Bruker AXS Inc., Madison, Wisconsin, USA.

[bb2] Ko, S.-K., Yang, Y.-K., Tae, J. & Shin, I. (2006). *J. Am. Chem. Soc.* **128**, 14150–14155. 10.1021/ja065114a 17061899

[bb3] Lakowicz, J. R. (2006). **TITLE?** 3rd ed., p. 67. New York:Springer.

[bb4] Sheldrick, G. M. (2008). *Acta Cryst.* A**64**, 112–122. 10.1107/S0108767307043930 18156677

[bb5] Wu, D., Huang, W., Duan, C.-Y., Lin, Z.-H. & Meng, Q.-J. (2007). *Inorg. Chem.* **46**, 1538–1540. 10.1021/ic062274e 17266305

[bb6] Zhang, L.-Z., Peng, X.-J., Gao, S. & Fan, J.-L. (2008). *Acta Cryst.* E**64**, o403. 10.1107/S1600536807068742 PMC2960358 21201431

